# New, Shorter Small‐Sample Intervals for Vaccine Efficacy

**DOI:** 10.1002/pst.70077

**Published:** 2026-02-18

**Authors:** Mauro Gasparini, Vincenzo Di Trani, Marco Ratta

**Affiliations:** ^1^ Department of Mathematical Sciences “G.L. Lagrange” Politecnico di Torino Torino Italy

**Keywords:** Bayesian methods, incidence rate, small‐sample asymptotics, surveillance times

## Abstract

In this work we illustrate a method to improve estimation of Vaccine Efficacy (VE), a vastly employed measure of effect in vaccine clinical research, with small and medium sample sizes. We introduce a comprehensive Bayesian approach that improves upon existing methodologies, explicitly considering patient recruitment processes to inform parameter estimation. In particular—in contrast to most methods currently used—in our proposed methodology the total number of cases as well as the censored surveillance times are seen as informative statistics, with underlying distributions which are used to derive the full likelihood. It turns out that our model depends on first and second moments of the surveillance times regardless of the recruitment process and, for finite sample sizes, it improves on the maximum likelihood method, which depends only on the first moment. The methodology is validated through extensive numerical simulations, demonstrating substantial improvements in parameter interval estimation across diverse scenarios and under multiple recruitment plans, when the number of events—and roughly the sample sizes—are small to medium. For large sample sizes, our method is equivalent to maximum likelihood. Markov Chain Monte Carlo (MCMC) simulations are needed and can be conducted very efficiently, due to an appropriate parameterization.

## 1 Introduction

How informative are the recruitment process and the resulting surveillance times when estimating Vaccine Efficacy (VE from now on)? This question is motivated by reconsideration of the methods used in two articles [1, 2] describing the 2020 pivotal clinical trial of the anti‐Covid‐19 vaccine sponsored by Pfizer/BioNTech: a conjugate Bayesian analysis or, respectively, the Clopper‐Pearson method are used there conditionally on the total number of cases and the surveillance times. This means that the observed total number of cases and the surveillance times were treated as constants and not incorporated in the methodology as informative realizations of random variables. These motivating examples give an opportunity to discuss the implications of the patient recruitment process—which affects surveillance times—in clinical trials and, more generally, to investigate up to what point it is useful, in a Bayesian analysis, modeling uncertain observable quantities related to the time dimension of the study.

Vaccine Efficacy, a central *estimand* in clinical vaccine research [3, 4], is a measure of the reduction in risk of developing the disease in the experimental treatment group when compared to a control group (which may be either placebo or another vaccine). Ewell [5] gave a review of several methods to calculate statistical intervals for VE, including Maximum Likelihood (ML), a Bayesian approach with conjugate priors (“method A”) and a frequentist method based on the exact binomial distribution (“method C”). A hybrid approach merging the latter two methods is the “exact method conditional on the total number of cases” [1]: the binomial distribution of the number of infected patients in the treatment group conditional on data is matched with a beta conjugate prior equivalent to the above mentioned “method A” to obtain credible intervals for VE. While a critical analysis of this and other approaches to the VE estimation of anti‐Covid‐19 vaccines is presented elsewhere [6, 7, 8], in this work we aim to improve the Bayesian approach by modeling surveillance times more properly.

The exact method conditional on the total number of cases is only an approximate Bayesian approach, since it makes only partial use of the full likelihood and of the Bayesian updating mechanism. In particular, the total number of cases and the surveillance times of the vaccinated and of the control cohorts are treated as known parameters instead of observed statistics, hence the adjective “conditional”: the method is a partially Bayesian method conditionally on the total number of cases and on the surveillance times.

This work contains a more complete full Bayesian approach which accounts for the joint distribution of the number of cases and the resulting surveillance times, clarifying the dependence of their joint distribution of the first and second moments of the surveillance times. This way, a more precise small sample estimation of VE is made possible.

The remaining part of the manuscript is divided as follows: in Section 2, VE estimation and the exact method conditional on the total number of cases are described, in Section 3 the full‐likelihood Bayesian model is derived, in Section 4 a simulation study is performed to validate the proposed approach under different recruitment processes and in Section 5 the Pfizer/BioNTech anti‐Covid‐19 vaccine data are re‐analyzed. Finally, conclusions are drawn in Section 6.

## 2 Current Statistical Models for VE

### 2.1 The Representation of Infection Processes as Point Processes

In Epidemiology, it is current practice to define the *incidence rate* as the ratio between the “number of new cases of disease or injury during a specified period” and the “time each person was observed, totaled for all persons” [9]. More particularly, a new infection process is obtained by stringing together one after the other all time periods participants have been exposed to the possibility of disease infection up until the earliest of the following four endpoints happens: onset of disease, death, loss to follow‐up, or end of study. The sum of the lengths of all time periods is the total surveillance time, while each participant experiencing the onset of disease contributes an event to the infection process; the other cases only contribute to the surveillance time, since we assume all other cases are non‐informatively censored observations.

In the context of a clinical trial, statistical modeling is complicated by the fact that a clinical trial takes place over a finite period of time. This creates censoring and makes it literally not true that the infection point process resulting from the stringing operation described above is homogeneous Poisson, although that may be a good approximation. A simple way to solve the problem and cast it using precise statistical modeling is to assume that, once recruited, a patient will be infected in an exponential time unless it is censored by end of study, unrelated death, or noninformative loss to follow‐up. In other words, the observed infection times are a random sample of randomly censored exponential times. The consequences of such assumptions are explored in Section 3.1.

In a standard comparative vaccine clinical trial there are two infection processes: one for the treatment group and an one for the control group. Let the parameters of the two exponential distributions be $\lambda_{c}$ for the control group and $\lambda_{v}$ for the vaccine group, each of them reflecting the intensity of the infection process in a stationary situation (i.e., without relevant peaks of contagion).

A common measure of comparison between the two infection processes is then the *incidence rate ratio*

$$\mathrm{IRR}=\frac{\lambda_{v}}{\lambda_{c}}$$

based on which VE is then defined as

(1) $$\mathrm{VE}=1-\mathrm{IRR}=1-\frac{\lambda_{v}}{\lambda_{c}}$$

which can be interpreted, in the usual case $\lambda_{v}<\lambda_{c}$, as the average fraction of missed infections (i.e., 100 × VE is the percentage of not infected vaccinated participants who would have been infected if not vaccinated).

Define

- $n_{v}=$ sample size of the vaccine group,
- $n_{c}=$ sample size the control group.

Then the data is composed of four basic statistics:

- $S_{v}=$ surveillance time of the vaccine group [person‐years],
- $S_{c}=$ surveillance time of the control group [person‐years],
- $X_{v}=$ number of infections in the vaccine group,
- $X_{c}=$ number of infections in the control group.

In our setup, the maximum likelihood estimator of IRR is, expressed in [person‐years^−1^]

(2) $$\hat{\mathrm{IRR}}=\frac{X_{v}\;S_{c}}{X_{c}\;S_{v}}.$$

Turning to logarithms and applying the delta method to our two samples of censored exponential observations, we obtain the approximate asymptotic distribution:

(3) $$\log\left(\hat{\mathrm{IRR}}\right)=\log\left(\frac{X_{v}\;S_{c}}{X_{c}\;S_{v}}\right)∼\text{Normal}\left(\log\left(\frac{\lambda_{v}}{\lambda_{c}}\right),\frac{1}{X_{v}}+\frac{1}{X_{c}}\right)$$

where the variance term has been approximated by its maximum likelihood estimator. In this work we refine these results by taking a deeper look into the structure of the stringed up process, then we construct the resulting Bayesian credible intervals for VE. First, in the next section we provide a brief review of the current intervals for VE.

### 2.2 Existing Statistical Intervals for Vaccine Efficacy

Many intervals for VE have appeared in the literature [5]. First comes the approximate maximum likelihood interval obtained from distribution (3):

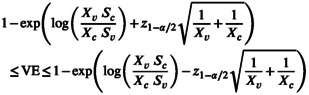

where $z_{1-\alpha /2}$ is the standard normal 1−α/2 quantile. This is method B of Ewell (1996) [5].

More recently, different intervals have been used, for example in the two Pfizer/BioNTech papers [1, 2]. In particular, the “exact method conditional on the total number of cases” [1] consists of assuming that the stringed up process is approximately Poisson and deriving as likelihood a binomial density for $X_{c}$ given $X_{v}+X_{c}$:

(4) xv+xcxvsvλvsvλv+scλcxv1−svλvsvλv+scλcxc

where we notice that the conditional probability of infection in the vaccine cohort is

(5) $$\theta =\frac{s_{v}\lambda_{v}}{s_{v}\lambda_{v}+s_{c}\lambda_{c}}=\frac{s_{v}\left(1-\mathrm{VE}\right)}{s_{v}\left(1-\mathrm{VE}\right)+s_{c}}.$$

In this approach, the observed surveillance times $s_{v}$ and $s_{c}$ are treated as constants and are used to “adjust” the estimate of VE according to Equation (5).

An explicit conditional Bayesian approach is followed: a conjugate beta prior, centered around a conservative estimate of VE, is given to the infection parameter θ. The second parameter of the prior is set to 1 to allow for the highest possible prior uncertainty, avoiding at the same time bimodality. The result is that a posteriori

(6) $$\theta ∼\text{Beta}\left(\frac{\hat{\theta }}{1-\hat{\theta }}+x_{v},1+x_{c}\right)$$

where $\hat{\theta }=\left(1-\hat{\mathrm{VE}}\right)/\left(2-\hat{\mathrm{VE}}\right)$ and $\hat{\mathrm{VE}}$ is a conservative estimate of VE. Once the posterior is obtained, the following $\left(1-\alpha \right)$‐level Bayesian *credible interval* can be computed regarding θ:

(7) $${\text{Beta}}_{\alpha /2}\left(\frac{\hat{\theta }}{1-\hat{\theta }}+x_{v},1+x_{c}\right)\leq \theta \leq {\text{Beta}}_{1-\alpha /2}\left(\frac{\hat{\theta }}{1-\hat{\theta }}+x_{v},1+x_{c}\right)$$

where ${\text{Beta}}_{\alpha /2}\left(n,m\right)$ is the α/2 quantile of a $\text{Beta}\left(n,m\right)$ distribution. A *ad‐hoc* “adjustment” is that the observed values $s_{v}$ and $s_{c}$ have to be plugged in Equation (5), while in the prior elicitation leading to $\hat{\theta }$ they are assumed to be equal, since prior to sampling they are not yet observed. This conceptual difficulty is addressed in Section 3.1.

Notice that a slight variation of the exact method conditional on the total number of cases amounts to giving an improper prior to θ and to obtain the posterior credible interval:

(8) $${\text{Beta}}_{\alpha /2}\left(x_{v},x_{c}\right)\leq \theta \leq {\text{Beta}}_{1-\alpha /2}\left(x_{v},x_{c}\right)$$

which, for moderate $x_{v}$ and $x_{c}$, is indistinguishable from the previous interval and avoids the above‐mentioned nuisance.

Finally, the second Pfizer/BioNTech paper [2] reports yet another interval: the binomial likelihood (4) is used to obtain the following $\left(1-\alpha \right)$‐level *Clopper‐Pearson (not‐Bayesian) exact* confidence interval

(9) $${\text{Beta}}_{\alpha /2}\left(x_{v},1+x_{c}\right)\leq \theta \leq {\text{Beta}}_{1-\alpha /2}\left(1+x_{v},x_{c}\right)$$

which, for moderate $x_{v}$ and $x_{c}$, is indistinguishable from the previous two intervals and avoids the above‐mentioned nuisance and any reference to a prior guess $\hat{\theta }$, being the result of a frequentist procedure which preserves at least the nominal level in an exact fashion.

The two Bayesian credible intervals and the last Clopper‐Pearson frequentist conservative interval can be worked backward to obtain corresponding intervals for VE itself:

(10) $$\frac{\left(1-H\right)s_{v}-Hs_{c}}{\left(1-H\right)s_{v}}\leq \mathrm{VE}\leq \frac{\left(1-L\right)s_{v}-Ls_{c}}{\left(1-L\right)s_{v}}$$

where L and H are the left‐hand (resp. right‐hand) side of Equations ((7), (8), (9)), respectively. Again, for moderate $x_{v}$ and $x_{c}$, the three intervals are basically the same, and we stress here that all of them are obtained *without any modelling of the recruitment process*. The goal of this work is to go beyond such limitation in order to make the most out of the information contained in the data, and specifically in the recruitment process, as illustrated in the next section.

## 3 The Full‐Likelihood Bayesian Approach

### 3.1 Motivation for the Full‐Likelihood Bayesian Approach

Consider a 1:1 randomization ratio in a clinical trial which goes on for a long time: the ratio between the control surveillance time $S_{c}$ and the vaccinated surveillance time $S_{v}$ approximates then the ratio of the two mean times to infection, by the law of large numbers applied once to the numerator and once to the denominator of the ratio. By definition, the ratio of the two mean times is 1‐VE, since the times to infection are exponentially distributed with mean $1/\lambda_{v}$ for the vaccinated and $1/\lambda_{c}$ for the control groups. Hence, the ratio of the surveillance times contains some additional information on VE, in addition to the number of cases in the two groups.

In this paper, we would like to take into account the surveillance times $S_{v}$ and $S_{c}$ in a more formal way than just plugging in their observed values in Equation (4). To do so, in the next section we derive a joint asymptotic distribution for all four relevant statistics $S_{v},S_{c},X_{v},X_{c}$.

### 3.2 A Bivariate Convergence Theorem

Since the vaccine and the control group give rise to independent observations, the joint distribution of $\left(S_{v},X_{v}\right)$ (resp. $\left(S_{c},X_{c}\right)$) is treated in this section using the unified notation $\left(S_{v},X_{v}\right)$, that is, dropping the subscripts, since the same results are true for the vaccine and the control group.

**FIGURE 1:**
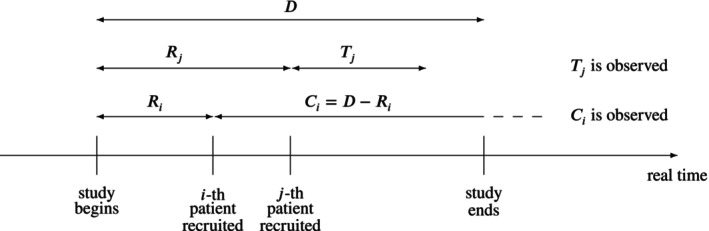
Random recruitment and censoring: The ith patient is censored, the jth patient is not.

Let D be the study duration in real time (in years) and let $R_{i}$ be the random recruitment time (in years) after study starts (and before study ends) and let $T_{i}$ be the time from recruitment to infection of the i−th patient: then, if the patient does not drop out before the end of the study, $C_{i}=D-R_{i}$ is the random censoring time (study duration D being a fixed quantity). Each patient contributes to the total surveillance time with an observed duration equal to the minimum of $T_{i}$ and $C_{i}$. Similarly, the i‐th patient increases the number of infections by 1 if $T_{i}<C_{i}$, and by 0 otherwise. Figure 1 is a graphical rendering. The total surveillance time and the number of infections are therefore sums of many i.i.d. random variables:

(11) $$S=\sum_{i=1}^{n}\min\left\{T_{i},C_{i}\right\}$$

(12) $$X=\sum_{i=1}^{n}\left(T_{i}<C_{i}\right)$$

where $\left(T_{i}<C_{i}\right)$ is the indicator function of the stated inequality. By the bivariate central limit theorem, the following holds.

Theorem 1 *Let* D *be the vaccine trial duration (constant) and, for the* i−t*h patient out of* n *patients, let* $T_{i}$ *be the random time from recruitment to infection*, $R_{i}$ *the random recruitment time and* $C_{i}=D-R_{i}$ *the random censoring time. Then, using definitions* (11) *and* (12) *and under the following assumptions:*

1. *times to infection* $T_{i},i=1,\ldots ,n$ *are, like* T, *i.i.d. exponential variables conditionally on their parameter* λ;
2. *censoring times* $C_{i},i=1,\ldots ,n$ *are i.i.d. like* C, *and independent of* $T_{i}$;

(13) $$\frac{1}{\sqrt{n}}\left(\begin{array}{c} S-n\;I_{1}\\ X-\mathrm{n\lambda}I_{1} \end{array}\right)\to N_{2}\left(\left(\begin{array}{c} 0\\ 0 \end{array}\right),\left(\begin{array}{cc} 2I_{2}-I_{1}^{2} & \lambda I_{2}-\lambda I_{1}^{2}\\ \lambda I_{2}-\lambda I_{1}^{2} & \lambda I_{1}-\lambda^{2}I_{1}^{2} \end{array}\right)\right)$$

*where*

(14) $$I_{1}=\int_{0}^{\infty }e^{-\mathrm{\lambda t}}\;P\left(C>t\right)\mathrm{dt}$$

(15) $$I_{2}=\int_{0}^{\infty }t\;e^{-\mathrm{\lambda t}}\;P\left(C>t\right)\mathrm{dt}$$

*as* n→∞ *the following convergence in distribution to a bivariate normal distribution holds:*

Proof is a consequence of the multivariate central limit theorem, by which

$$\frac{1}{\sqrt{n}}\left(\begin{array}{c} S-n\;E\left(\min\left(T,C\right)\right]\\ X-n\;E\left(\left(T<C\right)\right) \end{array}\right)\to N_{2}\left(\left(\begin{array}{c} 0\\ 0 \end{array}\right),\left(\begin{array}{cc} \mathrm{Var}\left(\min\left(T,C\right)\right) & \text{Covar}\left(\min\left(T,C\right),\left(T<C\right)\right)\\ \text{Covar}\left(\min\left(T,C\right),\left(T<C\right)\right) & \mathrm{Var}\left(\left(T<C\right)\right) \end{array}\right)\right)$$

Now, since both T and C are almost surely positive and independent,

$$\begin{array}{c} E\left(\min\left(T,C\right)\right)=\int_{0}^{\infty }P\left(\min\left(T,C\right)>t\right)\mathrm{dt}=\int_{0}^{\infty }P\left(T>t\right)\;P\left(C>t\right)\mathrm{dt}=\int_{0}^{\infty }e^{-\mathrm{\lambda t}}\;P\left(C>t\right)\mathrm{dt}=I_{1}\\ E\left(\min{\left(T,C\right)}^{2}\right)=\int_{0}^{\infty }P\left(\min{\left(T,C\right)}^{2}>t\right)\mathrm{dt}=\int_{0}^{\infty }P\left(T>\sqrt{t}\right)\;P\left(C>\sqrt{t}\right)\mathrm{dt}=2\int_{0}^{\infty }t\;e^{-\mathrm{\lambda t}}\;P\left(C>t\right)\mathrm{dt}=2I_{2} \end{array}$$

and the first variance can be obtained as follows:

$$\mathrm{Var}\left(\min\left(T,C\right)\right)=E\left[\min{\left(T,C\right)}^{2}\right]-E{\left[\min\left(T,C\right)\right]}^{2}=2I_{2}-I_{1}^{2}.$$

Next, since $\left(T<C\right)$ is an indicator function, that is, a Bernoulli variate with probability parameter π,

(16) $$\pi =E\left(\left(T<C\right)\right)=P\left(T<C\right)=\int_{t<c}\lambda e^{-\mathrm{\lambda t}}f_{C}\left(c\right)\text{dcdt}=\lambda \int_{0}^{\infty }e^{-\mathrm{\lambda t}}\;P\left(C>t\right)\mathrm{dt}=\lambda I_{1}$$

and finally

$$E\left(\min\left(T,C\right)\cdot \left(T<C\right)\right)=\int_{0}^{\infty }t\;\lambda \;e^{-\mathrm{\lambda t}}\;P\left(C>t\right)\;\mathrm{dt}=\lambda I_{2}$$

and the theorem is proved by calculating

$$\begin{array}{c} \text{Covar}\left(\min\left(T,C\right),\left(T<C\right)\right)\\ \;=E\left(\min\left(T,C\right)\cdot \left(T<C\right)\right)-E\left(\min\left(T,C\right)\right)E\left(\left(T<C\right)\right)=\lambda I_{2}-\lambda I_{1}^{2}. \end{array}$$

It should be stressed that the Theorem contains an asymptotic distribution of S and X which depends on recruitment process, but only through the first and second moments of the surveillance times. Therefore, in this approach any kind of recruitment can be accommodated, including multi‐center trials, recruitments with staggered entry of the patients and trials with recruitment stopped before the end of the trial.

Notice also that convergence in formula (3) can be recovered as a by‐product from convergence in formula (13) by applying the delta method. This has several important implications:

- The result in the theorem is an extension of the usual large‐sample approximation theorem for the maximum likelihood estimator;
- Optimality of the ML method for our regular model provides a reference (e.g., in terms of width of interval estimation for VE, both frequentist and Bayesian) which cannot be beaten asymptotically; however, with our more comprehensive results, there is hope to improve finite sample size results, in the spirit of small‐sample asymptotics;
- While convergence in Formula (3) involves the first moment of the surveillance times (hidden in the variance), the bivariate approach in Theorem 1 involves the first and second moments of the surveillance times, but no other details regarding recruitment.

### 3.3 The Full‐Likelihood Bayesian Model

Applying now the results of the theorem to both control and vaccinated groups, we can write the likelihood of the unknown parameters, having observed $S_{c}=s_{c},X_{c}=x_{c},S_{v}=s_{v},X_{v}=x_{v}$, in the following way:
(17)

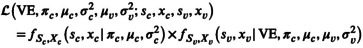

where

$$\begin{array}{c} \mathrm{VE}=1-\frac{\lambda_{v}}{\lambda_{c}}\\ \pi_{c}=P\left(T_{c}<C\right)=\lambda_{c}I_{1,c}\\ \mu_{c}=I_{1,c}=\int_{0}^{\infty }e^{-\lambda_{c}t}\;P\left(C>t\right)\mathrm{dt}\\ \sigma_{c}^{2}=2I_{2,c}-I_{1,c}^{2}=\int_{0}^{\infty }t\;e^{-\lambda_{c}t}P\left(C>t\right)\mathrm{dt}-I_{1,c}^{2}\\ \mu_{v}=I_{1,v}=\int_{0}^{\infty }e^{-\lambda_{v}t}\;P\left(C>t\right)\mathrm{dt}\\ \sigma_{v}^{2}=2I_{2,v}-I_{1,v}^{2}=\int_{0}^{\infty }t\;e^{-\lambda_{v}t}\;P\left(C>t\right)\mathrm{dt}-I_{1,v}^{2} \end{array}$$

and $f_{S_{c},X_{c}}\left(\cdot \right)$ and $f_{S_{c},X_{c}}\left(\cdot \right)$ are, respectively, the density functions of the following asymptotic bivariate normal distributions

$$\begin{array}{c} \left(\begin{array}{c} S_{c}\\ X_{c} \end{array}\right)∼N_{2}\left(\left(\begin{array}{c} n_{c}I_{1,c}\\ n_{c}\lambda_{c}I_{1,c} \end{array}\right),n_{c}\left(\begin{array}{cc} 2I_{2,c}-I_{1,c}^{2} & \lambda_{c}I_{2,c}-\lambda_{c}I_{1,c}^{2}\\ \lambda_{c}I_{2,c}-\lambda_{c}I_{1,c}^{2} & \lambda_{c}I_{1,c}-\lambda_{c}^{2}I_{1,c}^{2} \end{array}\right)\right)\\ \left(\begin{array}{c} S_{v}\\ X_{v} \end{array}\right)∼N_{2}\left(\left(\begin{array}{c} n_{v}I_{1,v}\\ n_{v}\lambda_{v}I_{1,v} \end{array}\right),n_{v}\left(\begin{array}{cc} 2I_{2,v}-I_{1,v}^{2} & \lambda_{v}I_{2,v}-\lambda_{v}I_{1,v}^{2}\\ \lambda_{v}I_{2,v}-\lambda_{v}I_{1,v}^{2} & \lambda_{v}I_{1,v}-\lambda_{v}^{2}I_{1,v}^{2} \end{array}\right)\right) \end{array}$$

**FIGURE 2:**
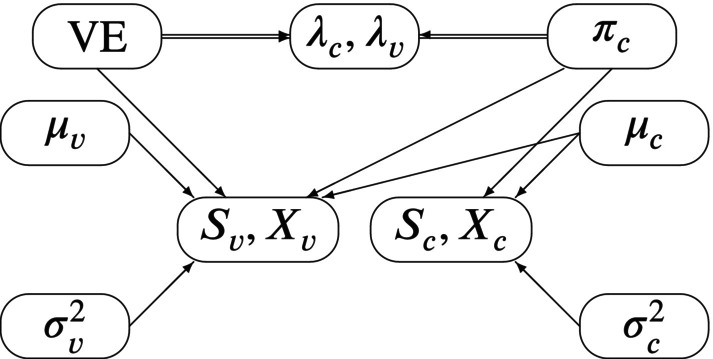
DAG for the full‐likelihood Bayesian model: Double arrows indicate the functional dependence of $\left(\lambda_{v},\lambda_{c}\right)$ on VE and $\pi_{c}$.

By adding priors to the parameters VE, $\pi_{c},\mu_{c},\sigma_{c}^{2},\mu_{v},\sigma_{v}^{2}$, the full‐likelihood Bayesian model is obtained and illustrated graphically by its associated Directed Acyclic Graph (DAG) in Figure 2.

### 3.4 Default Prior Elicitation

The reason for parameterizing the likelihood in terms of $\mathrm{VE},p_{c},\mu_{c},\sigma_{c}^{2},\mu_{v},\sigma_{v}^{2}$ (instead of, say, $\lambda_{c}$ and $\lambda_{v}$) is first to stress that VE is the main object of inference, but also to facilitate convergence of the MCMC algorithm used to approximate the Bayesian posterior inference mechanism: with this parameterization, all parameters except VE have distributions with bounded support, and in the total absence of information one could choose to give a uniform prior to each of them.

Regarding VE instead, in the literature it is common to center a prior on VE on a suitable prior guess $\hat{\mathrm{VE}}$, a typically low value, say $\hat{\mathrm{VE}}=0.30$, in order not to bias the estimation process towards optimistic values. Using the same idea of transforming to parameters with bounded support, a possible default prior for VE is therefore the one induced by

$$\frac{1-\mathrm{VE}}{2-\mathrm{VE}}∼\text{Beta}\left(1-\hat{\mathrm{VE}},1\right)$$

as done in the existing Bayesian literature on VE [1]. For the other parameters, independently,

$$\begin{array}{c} p_{c}∼\text{Uniform}\left(0,1\right)\\ \mu_{c}∼\text{Uniform}\left(0,D\right)\\ \sigma_{c}^{2}∼\text{Uniform}\left(0,D^{2}\right)\\ \mu_{v}∼\text{Uniform}\left(0,D\right)\\ \sigma_{v}^{2}∼\text{Uniform}\left(0,D^{2}\right). \end{array}$$

One last trick to enhance convergence and stability of the MCMC algorithm is to simulate $\left(S_{c},X_{c}\right)$ and $\left(S_{v},X_{v}\right)$ first by simulating $X_{c}$ (resp. $X_{v}$) from its exact binomial distribution, then $S_{c}$ (resp. $S_{v}$) from the conditional normal distribution implied by the bivariate normal asymptotic distribution.

## 4 Numerical Results

In this section, the proposed full likelihood Bayesian (FB) approach is compared to the other intervals used to estimate VE. The simulation setting is introduced in Section 4.1 as well as the evaluation metrics employed, while the results are discussed in Section 4.2.

### 4.1 Simulation Setting

For the simulation study presented hereinafter, clinical trials with a fixed duration D=1 are considered. The infection rate in the control group is set to $\lambda_{c}=0.1$, which is of the same order of magnitude as that observed during the COVID‐19 pandemic. All other free parameters are varied to evaluate the performance of the proposed approach under a broad range of plausible scenarios.

The true vaccine efficacy (VE) is varied between 0.1 and 0.9 in increments of 0.2, to reflect different efficacy regimens. Accordingly, the infection rate in the vaccine group is defined as $\lambda_{v}=\left(1-\mathrm{VE}\right)\lambda_{c}$.

The data are simulated from two different recruitment processes:

- Uniform recruitment: subjects are recruited at i.i.d. times like $R∼U\left(0,\mathrm{\tau D}\right)$, that is, according to an accrual process with constant rate for a fraction τ of the trial duration D, so that the density of the infection time R is
$$f_{R}\left(r\right)=\frac{1}{\mathrm{\tau D}}\;\;r\in \left(0,\mathrm{\tau D}\right);$$
- Beta recruitment: subjects are recruited at i.i.d. times according to a rescaled Beta(2,2) distribution over the interval $\left(0,\mathrm{\tau D}\right)$, representing an accrual process with increasing rate in the early phase and decreasing rate in the later phase of the trial. Specifically, the density of the infection time R is as follows:
$$f_{R}\left(r\right)=6\;\frac{r}{\mathrm{\tau D}}\;\left(1-\frac{r}{\mathrm{\tau D}}\right)\;\;r\in \left(0,\mathrm{\tau D}\right).$$

In both recruitment schemes, τ=0.75, implying that recruitment occurs during the first 75% of the trial duration. This choice ensures each subject a minimum time of 3 months for being exposed to infection.

For each combination of VE and recruitment scheme, the total sample size $n_{c}+n_{v}$ is calculated to achieve a target expected number of total infections $E\left(X_{c}+X_{v}\right)\in \left\{40,80,160,900\right\}$ across the two arms. Assuming a 1:1 allocation ratio between the vaccine and control groups, the sample size is approximated using the following formula:

$$n_{c}=n_{v}=\frac{E\left(X_{c}+X_{v}\right)}{\pi_{c}+\pi_{v}},$$

where

$$\begin{array}{c} \pi_{c}=1-\int_{0}^{\mathrm{\tau D}}e^{-\lambda_{c}\left(D-r\right)}f_{R}\left(r\right)\mathrm{dr},\\ \pi_{v}=1-\int_{0}^{\mathrm{\tau D}}e^{-\lambda_{v}\left(D-r\right)}f_{R}\left(r\right)\mathrm{dr}. \end{array}$$

In the data generation process, for each patient i, the recruitment time $R_{i}$ is sampled from the chosen recruitment distribution (Uniform or Beta), and the time to infection $T_{i}$ is sampled from an exponential distribution with rate $\lambda_{c}$ (resp. $\lambda_{v}$). Total surveillance times and infection counts per group are then computed according to Equations (11) and (12), respectively.

For each simulation scenario, 10,000 datasets are generated. An approximation of the posterior distribution of VE is obtained via Markov Chain Monte Carlo (MCMC) sampling. Specifically, three MCMC chains of 20000 iterations each are run per dataset, with a burn‐in of 2000 iterations and a thinning factor of 10. Sampling is performed using the JAGS software [10], interfaced through the R environment [11].

### 4.2 Results

**FIGURE 3:**
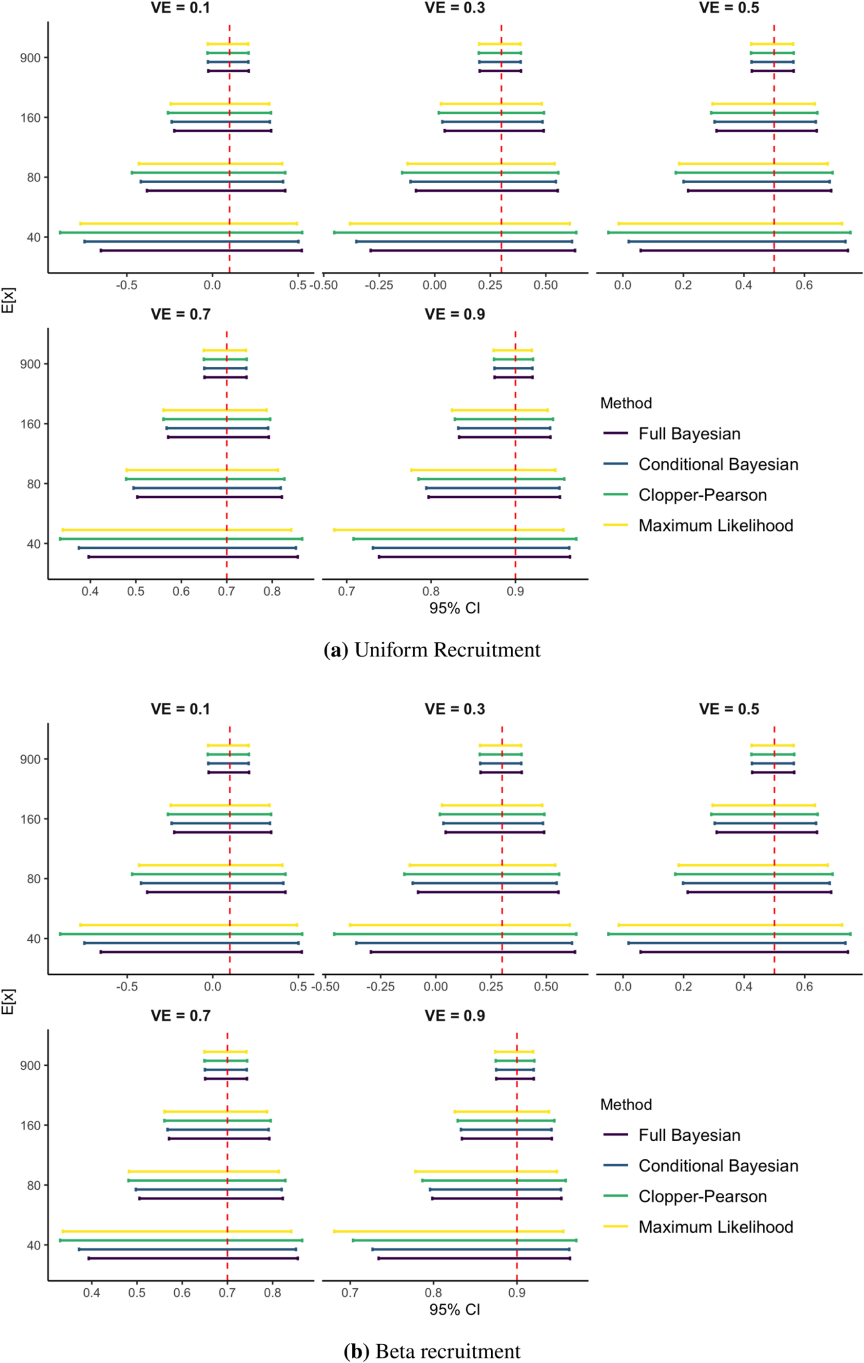
Comparison of the 95% intervals for VE.

The performance of our new full likelihood Bayesian (FB) approach is assessed using the following metrics:

- *Average coverage of the 95% credibility or confidence intervals* for each method, to evaluate *frequentist calibration* [12, 13] and lack of excessive influence of the prior distributions:
- *Average percentage reduction in interval width* of the 95% intervals for VE when using FB, compared to competing methods, to assess *interval precision*;

The results of the simulation study for the two recruitment processes considered are summarized in Table 1 (uniform recruitment) and Table 2 (Beta recruitment) and rendered graphically in Figure 3.

**TABLE 1:** Comparison between the full likelihood Bayesian and the other approaches: Uniform recruitment.

VE	R	$E\left(X_{c}+X_{v}\right)$	$n_{c}+n_{v}$	% Coverage				% Width reduction		
				FB	CB	CP	ML	CB	CP	ML
0.1	Uniform	40	697	94.8	94.9	96.3	95.5	5.52	16.08	6.93
0.1	Uniform	80	1393	95.2	95.1	96.4	95.3	2.69	9.62	3.50
0.1	Uniform	160	2786	94.6	94.6	95.6	94.9	1.20	5.93	1.72
0.1	Uniform	900	15,668	94.6	94.7	95.1	94.8	−0.11	1.83	0.11
0.3	Uniform	40	777	95.2	95.2	96.8	95.8	4.85	14.96	6.91
0.3	Uniform	80	1553	94.9	95.0	96.3	95.5	2.32	9.10	3.49
0.3	Uniform	160	3105	94.8	94.9	95.8	95.2	1.14	5.76	1.80
0.3	Uniform	900	17,465	95.1	95.1	95.5	95.2	−0.12	1.84	0.15
0.5	Uniform	40	879	95.0	95.1	96.6	95.7	4.09	14.00	7.26
0.5	Uniform	80	1757	95.0	95.2	96.4	95.5	1.97	8.69	3.67
0.5	Uniform	160	3514	95.2	95.1	96.1	95.3	0.92	5.58	1.86
0.5	Uniform	900	17,465	95.1	95.1	95.5	95.2	−0.12	1.84	0.15
0.7	Uniform	40	1014	95.0	94.8	96.6	95.7	3.41	13.41	8.76
0.7	Uniform	80	2028	95.2	95.1	96.6	95.3	1.66	8.62	4.45
0.7	Uniform	160	4055	95.2	95.2	96.3	95.5	0.79	5.71	2.28
0.7	Uniform	900	22,808	95.3	95.4	95.8	95.5	−0.01	2.18	0.42
0.9	Uniform	40	1202	95.7	95.5	97.7	96.0	2.57	14.53	17.80
0.9	Uniform	80	2403	95.3	95.3	97.1	95.6	1.28	9.10	9.17
0.9	Uniform	160	4806	94.9	94.8	96.2	94.9	0.63	7.07	4.71
0.9	Uniform	900	27,030	94.9	94.9	95.5	94.9	−0.01	2.96	0.87

Abbreviations: CB, conditional Bayesian; CP, Clopper‐Pearson frequentist; FB, full likelihood Bayesian; ML, maximum likelihood frequentist.

**TABLE 2:** Comparison between the full likelihood Bayesian and the other approaches: Rescaled beta recruitment.

VE	R	$E\left(X_{c}+X_{v}\right)$	$n_{c}+n_{v}$	% Coverage				% Width reduction		
				FB	CB	CP	ML	CB	CP	ML
0.1	Beta	40	696	95.0	94.9	96.4	95.6	5.59	16.08	6.94
0.1	Beta	80	1391	95.1	95.1	96.3	95.5	2.73	9.65	3.53
0.1	Beta	160	2782	95.0	95.2	95.9	95.3	1.28	5.96	1.76
0.1	Beta	900	15,647	94.8	94.6	95.2	94.9	−0.15	1.82	0.09
0.3	Beta	40	776	95.2	95.2	96.7	95.9	4.95	15.05	6.99
0.3	Beta	80	1551	95.2	95.2	96.4	95.5	2.37	9.12	3.52
0.3	Beta	160	3101	95.0	95.2	96.0	95.4	1.07	5.71	1.76
0.3	Beta	900	17,443	95.2	95.1	95.6	95.2	−0.25	1.71	0.01
0.5	Beta	40	878	95.2	95.1	96.7	95.7	4.08	13.93	7.20
0.5	Beta	80	1755	95.1	95.0	96.3	95.5	1.98	8.71	3.69
0.5	Beta	160	3510	94.8	94.8	95.7	95.0	0.92	5.62	1.90
0.5	Beta	900	19,740	94.8	94.8	95.2	94.9	−0.21	1.82	0.12
0.7	Beta	40	1013	95.2	95.0	96.8	95.8	3.37	13.42	8.71
0.7	Beta	80	2025	95.4	95.1	96.4	95.5	1.58	8.59	4.42
0.7	Beta	160	4050	95.1	95.0	96.0	95.1	0.76	5.71	2.28
0.7	Beta	900	22,780	94.7	94.8	95.2	94.8	−0.01	2.17	0.41
0.9	Beta	40	1200	95.2	95.1	97.7	95.2	2.55	14.47	17.58
0.9	Beta	80	2400	95.4	95.3	97.1	95.9	1.30	10.04	9.23
0.9	Beta	160	4799	94.7	94.7	96.1	95.2	0.67	7.08	4.73
0.9	Beta	900	26,994	94.8	94.8	95.6	95.0	0.07	3.04	0.96

Abbreviations: CB, conditional Bayesian; CP, Clopper‐Pearson frequentist; FB, full likelihood Bayesian; ML, maximum likelihood frequentist.

Regarding the average coverage of the 95% credibility intervals, the Full Bayesian (FB) method consistently approaches the nominal level across all evaluated scenarios. A similar behavior is observed for the Conditional Bayesian (CB) approach. In contrast, the two frequentist methods—Clopper–Pearson (CP) and asymptotic maximum likelihood (ML) confidence intervals—exhibit average coverage consistently above the nominal level, confirming the conservative nature of both procedures, particularly when few events are observed and asymptotic assumptions may not hold. In light of these findings and of the small oscillations around the nominal level, all the methods considered can be regarded as well‐calibrated in the frequentist sense [12, 13]. In all configurations analyzed, the Monte Carlo Standard Error (MCSE) associated with the estimate of the coverage probability is approximately 0.2%.

When comparing the width of the 95% intervals for VE (either credibility or confidence, depending on whether the approach is Bayesian or frequentist), the FB method shows a uniform improvement over all competing methods across the considered scenarios. In particular, the reduction in interval width compared to all the other approaches is more substantial when a small to moderate number of infections is observed and gradually diminishes as the number of infections increases. Notably, the width of the FB credibility interval is very close to the width of the asymptotic ML interval when the expected number of infections is at least 900.

For a fixed expected number of infections, a larger average percentage reduction in the width of the FB credibility interval compared to the CB credibility interval is observed for lower values of VE, while a basic agreement between the two is observed when VE = 0.9 (recall that the prior distribution used for VE is the same in the two Bayesian approaches, hence the comparison between the two approaches is fair). When comparing the FB method with the two frequentist approaches, a comparable reduction in interval width is observed across different values of VE, particularly when VE is low to moderate. However, a notably larger average percentage reduction is observed for the FB method when VE equals 0.9. This result is consistent with the well‐documented tendency of both frequentist confidence intervals to become overly conservative at high values of VE. In all considered scenarios the Monte Carlo Standard Error (MCSE) associated with the estimate of the percentage reduction in interval width is < 0.1%.

No meaningful differences in the average percentage reduction in interval width are observed across the two recruitment processes (uniform and beta) considered.

## 5 The Pfizer/BioNTech Trial Revisited

In this section, we apply our methodology to the Pfizer/BioNTech trial [1, 2] of the antiCOVID‐19 vaccine which took place in 2020 and 2021. In Table 3, data related to the full trial population at different times and for different subgroups are reported. The analysis is carried out using the proposed full likelihood Bayesian (FB) approach, as well as the Conditional Bayesian (CB), the Clopper–Pearson (CP) procedure, and the asymptotic Maximum Likelihood (ML) method.

**TABLE 3:** Comparison of VE estimation with different Bayesian and frequentist approaches using published data [1, 2] regarding the Pfizer/Biontech Covid‐19 trial.

References	Subgroup	Data			Estimate of 100 × VE				% Width reduction		
		Control	Vaccine	Duration	FB	CB	CP	ML	CB	CP	ML
Thomas [2]	Overall March 2021	$\begin{array}{l} n_{c}=20713\\ x_{c}=850\\ s_{c}=6003 \end{array}$	$\begin{array}{l} n_{v}=20712\\ x_{v}=77\\ s_{v}=6247 \end{array}$	D=0.55	91.27 (89.07, 93.14)	91.26 (89.04, 93.13)	91.30 (89.00, 93.20)	91.30 (89.01, 93.11)	0.22	3.04	0.64
Polack [1]	Overall Nov. 2020	$\begin{array}{l} n_{c}=17511\\ x_{c}=162\\ s_{c}=2222 \end{array}$	$\begin{array}{l} n_{v}=17411\\ x_{v}=8\\ s_{v}=2214 \end{array}$	D=0.21	94.87 (90.38, 97.63)	94.83 (90.33, 97.63)	95.04 (90.00, 97.90)	95.04 (89.92, 97.56)	0.59	8.19	5.13
Polack [1]	Male	$\begin{array}{l} n_{c}=8762\\ x_{c}=81\\ s_{c}=1108 \end{array}$	$\begin{array}{l} n_{v}=8875\\ x_{v}=3\\ s_{v}=1124 \end{array}$	D=0.21	96.00 (89.82, 98.90)	95.93 (89.67, 98.89)	96.35 (88.94, 99.26)	96.35 (88.44, 98.85)	1.46	12.01	12.74
Polack [1]	Hispanic or Latinx	$\begin{array}{l} n_{c}=4746\\ x_{c}=53\\ s_{c}=600 \end{array}$	$\begin{array}{l} n_{v}=4764\\ x_{v}=3\\ s_{v}=605 \end{array}$	D=0.21	93.88 (84.18, 98.33)	93.78 (83.92, 98.29)	94.39 (82.68, 98.88)	94.39 (82.04, 98.25)	1.58	12.68	12.74
Polack [1]	Over 65	$\begin{array}{l} n_{c}=3880\\ x_{c}=19\\ s_{c}=511 \end{array}$	$\begin{array}{l} n_{v}=3848\\ x_{v}=1\\ s_{v}=508 \end{array}$	D=0.21	93.30 (73.17, 99.24)	92.92 (71.71, 99.22)	94.71 (66.70, 99.87)	94.71 (60.45, 99.29)	5.10	21.32	32.79
Polack [1]	Brazil	$\begin{array}{l} n_{c}=1121\\ x_{c}=8\\ s_{c}=117 \end{array}$	$\begin{array}{l} n_{v}=1129\\ x_{v}=1\\ s_{v}=119 \end{array}$	D=0.21	85.85 (38.09, 98.49)	84.30 (29.59, 98.36)	87.71 (8.33, 99.72)	87.71 (1.74, 98.46)	12.18	33.92	37.56

Abbreviations: CB, conditional Bayesian; CP, Clopper‐Pearson frequentist; FB, full likelihood Bayesian; ML, maximum likelihood frequentist.

Information on sample sizes, number of infections, and surveillance times for each cohort is publicly available [12]. Data regarding the maximum duration at risk (expressed in years) are obtained by subtracting 28 days from the total trial duration, which is calculated from the accrual start date (July 27, 2020) and the data cut‐off date (November 14, 2020 in Polack [1] and March 13, 2021 in Thomas [2]). This adjustment is due to the study protocol, which considers a participant at risk of infection starting 7 days after receiving the second vaccine dose (administered 21 days after randomization).

While all methods yield comparable point estimates of vaccine efficacy (VE) across the considered subgroups, notable differences arise in the width of the corresponding 95% intervals. In particular, the FB approach consistently provides narrower intervals, suggesting improved precision relative to the other methods. As detailed in Section 4, this advantage is most pronounced in smaller subgroups—that is, those with fewer observed infections—such as the Brazilian cohort and individuals aged over 65. In these subgroups, where the number of infections is approximately 10 and 20, respectively, the 95% credible intervals produced by the FB method are narrower by about 12% and 5% compared to CB, 33% and 21% compared to CP, and 37% and 32% compared to ML‐based confidence intervals. A more modest, yet still meaningful, improvement in precision is observed in subgroups with a moderate number of infections, such as individuals aged over 55 and the male subgroup analyzed in Polack [2]. In these cases, the FB method yields a reduction in interval width between 1.5% and 3.5% relative to CB, and between 12% and 14% relative to the frequentist approaches. Finally, all methods produce nearly identical interval widths when applied to the overall study population reported in March 2021 by Thomas [1], where the number of infections exceeds 900. This finding aligns with the simulation results in Section 4 and reflects the expected asymptotic convergence of the different procedures as the number of observed events increases.

## 6 Discussion

In this work, a novel Bayesian approach is proposed for the estimation of vaccine efficacy (VE). The main innovation lies in the joint modeling of the number of infections and surveillance times, derived through an asymptotic approximation based on the Bivariate Central Limit Theorem.

An extensive simulation study, exploring different VE scenarios, recruitment processes, and sample sizes, showed that the proposed approach yields narrower credibility intervals than standard Bayesian and frequentist methods, with no loss in coverage. This advantage is especially pronounced when the number of infections is low to moderate, making the method particularly useful in subgroup analyses or small‐to‐medium‐sized trials.

Our new approach does not require any specific distributional assumptions about the recruitment process, making it highly flexible. The first and second moments of the distribution of the surveillance times, which affect the asymptotic distribution of VE as illustrated in Theorem 1, can be easily estimated within a Bayesian framework outlined in Section 3.3 using the observed surveillance times, some of which may be censored. Therefore, our results apply to any recruitment scheme with exchangeable patients. The recruitment process itself does not need to be made explicit; therefore, our results are applicable in many common circumstances.

For simplicity, the simulation study focused on two stylized recruitment processes: a uniform process and a process with Beta infection times. However, only minor changes in performance are expected if more realistic recruitment processes are considered, such as those proposed by Senn [14] or Anisimov [15].

While an analytical posterior distribution for VE is available under the Conditional Bayesian approach (due to the beta‐binomial conjugacy), no closed‐form expression exists for the full likelihood Bayesian approach, which therefore requires the use of MCMC methods. Nevertheless, given the limited complexity of the model, posterior estimation remains highly efficient and does not pose any practical challenges.

Our results may have important consequences for the design of future vaccine trials of intermediate size (such as the ones discussed by Ewell [5]), especially regarding sample size determination and recruitment strategies.

## Funding

This work was supported by Ministero dell'Università e della Ricerca (D34H‐Digital Driven Diagnostics).

## Conflicts of Interest

The authors declare no conflicts of interest.

## Data Availability

Data sharing is not applicable to this article as no datasets were generated or analyzed during the current study.

## Appendix A JAGS Code

The data and the model of the JAGS code to reproduce the analysis of the Pfizer/BioNTech data are listed below:
